# The Malformations, Diseases, and Injuries of the Fingers and Toes, and Their Surgical Treatment

**Published:** 1866-04

**Authors:** 


The Malformations, Diseases, and Injuries of the Fingers and Toes, and their Surgical Treatment. By Thomas Annandale, F.R.C.S. Edinburgh; Lecturer on Surgery; Assistant-Surgeon to the Edinburgh Royal Infirmary. The Jacksonian Prize Essay for the year 1864. Philadelphia: J. B. Lippincott & Co. 1866.

This is an octavo volume of 292 pages, printed on excellent type and good paper, with a considerable number of well-executed plates and drawings for illustration. The work is divided into seven chapters, embracing the following topics, namely:—Congenital Affections; Inflammatory Affections; Tumors; Injuries; Non-Congenital Contractions and Distortions; Resections; and Amputations. The work is one of real merit, and constitutes a valuable addition to our surgical literature.

For sale by S. C. Griggs & Co., Lake St. Price $5.

